# Hemophagocytic Lymphohistiocytosis Secondary to Prostatic Adenocarcinoma

**DOI:** 10.7759/cureus.12798

**Published:** 2021-01-20

**Authors:** Pramuditha Rajapakse, Sushmita D Shrestha, Kamila Bakirhan

**Affiliations:** 1 Internal Medicine, Danbury Hospital, Yale School of Medicine, Danbury, USA; 2 Oncology, Danbury Hospital, Yale School of Medicine, Danbury, USA

**Keywords:** hemophagocytic lymphohistiocytosis (hlh), secondary hemophagocytic lymphohistiocytosis, prostatic adenocarcinoma

## Abstract

Hemophagocytic lymphohistiocytosis (HLH) is caused by excessive immune activation. It can be primary in the setting of genetic defects or secondary in the setting of infection, inflammation, and malignancy. Here we present the fourth reported case of secondary HLH in association with prostatic adenocarcinoma and the diagnostic challenges of this rare, life-threatening condition. This is a 78-year-old male who presented to the hospital with fever and generalized weakness for three days and found to have splenomegaly, pancytopenia, markedly elevated transaminases, and ferritin. Bone marrow biopsy revealed hemophagocytes. He underwent an extensive evaluation to identify the etiology. Para-aortic lymph node biopsy was consistent with prostatic adenocarcinoma. The diagnosis of HLH needs a high index of suspicion because the presentation is nonspecific. HLH is a rapidly progressive and potentially fatal condition underscoring the need for a prompt evaluation if this condition is suspected.

## Introduction

Hemophagocytic lymphohistiocytosis (HLH) is an aggressive hyperinflammatory syndrome induced by aberrantly activated macrophages and cytotoxic T cells. It can be primary due to genetic defects and secondary in the setting of infection, inflammation, and malignancy [1-3]. Most malignancies triggering secondary HLH are hematological malignancies including lymphomas and leukemia. Solid tumors are rarely associated with HLH [4]. Only three cases of prostate cancer-associated HLH have been documented in the literature thus far [5-7]. Here we present a case of secondary HLH associated with prostatic adenocarcinoma.

## Case presentation

**Figure 1:**
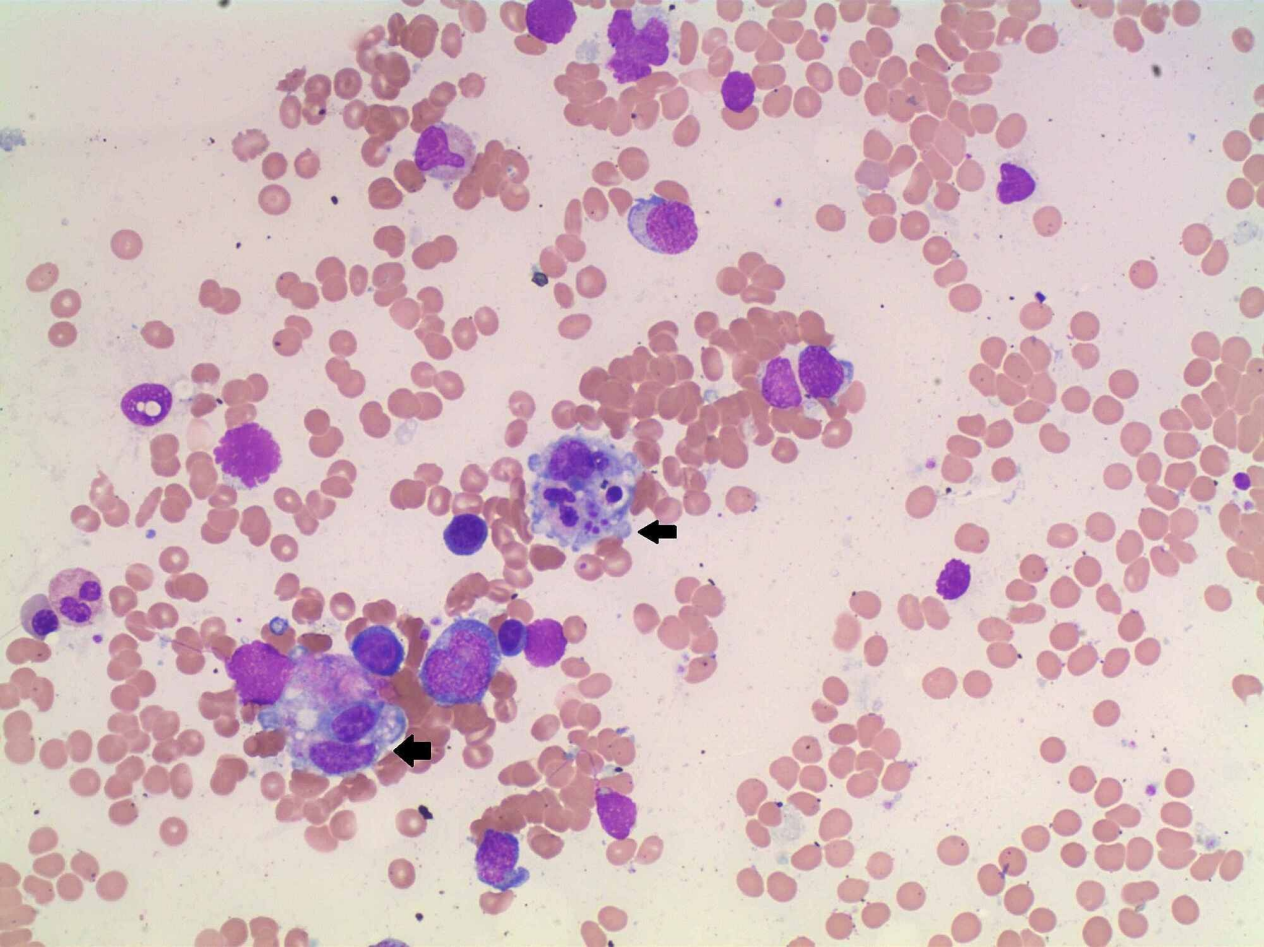
Bone marrow aspiration and biopsy: trilineage hematopoiesis with focal hemophagocytosis.

**Figure 2:**
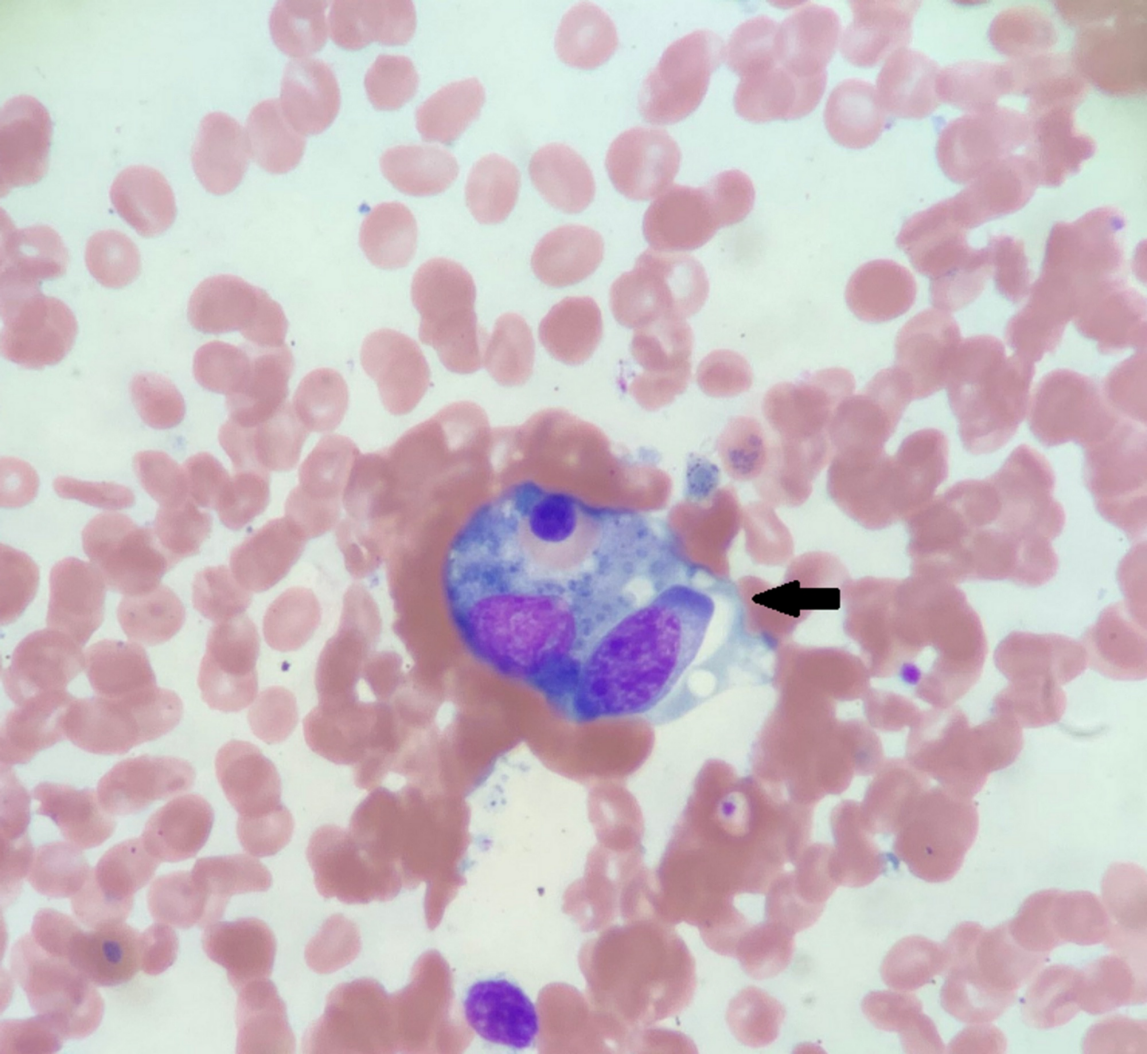
Bone marrow aspirate and biopsy demonstrating focal hemophagocytosis.

This is a 78-year-old male who presented to the hospital with fever and generalized weakness for three days. He was an avid golfer and was very functional and healthy before the onset of symptoms. On arrival at the Emergency Department, he was febrile with a temperature of 38.9°C and the remaining vital signs were unremarkable. Physical exam showed conjunctival pallor and splenomegaly. Initial laboratory evaluation revealed leukopenia with a WBC count of 0.7 x 10^9^/L (range 3.5 x 10^9^-10 x 10^9^/L), thrombocytopenia with a platelet count of 58 x 10^9^/L (150 x 10^9^-400 x 10^9^/L). A baseline complete blood count (CBC) was not available for comparison. Serum chemistry showed elevated creatinine at 2 mg/dL (0.67-1.23 mg/dL) and acute liver failure with alanine aminotransferase (ALT) and aspartate aminotransferase (AST) >7000 U/L (range for ALT 10-62 U/L, AST 10-50 U/L). Due to the concern of multiorgan failure and disseminated intravascular coagulation, the patient underwent infectious workup and coagulation studies. Blood cultures yielded no growth, and chest x-ray and urinalysis were unremarkable. However, prothrombin time was elevated at 15 seconds (range 12.4-14.5 seconds) and international normalized ratio (INR) was normal. Fibrinogen level was low at 100 mg/dL (210-480 mg/dL). The PLASMIC score was 4 indicating a low risk of severe disintegrin and metalloproteinase with a thrombospondin type 1 motif, member 13 (ADAMTS13) deficiency, and thus thrombotic thrombocytopenic purpura (TTP) was less likely. Additionally, there were no schistocytes noted in the peripheral blood smear. Within the first few days, pancytopenia and coagulopathy continued to worsen requiring multiple blood product transfusions. This made us consider atypical infections, possibly causing bone marrow suppression or malignancy. Further testing revealed an elevated ferritin level at 46,712 ng/mL (range 24-370 ng/mL). The differential diagnoses at this point were infection, macrophage activation syndrome (MAS), and primary liver failure. However, the extreme elevation of ferritin prompted us to consider HLH. Bone marrow aspiration and biopsy revealed trilineage hematopoiesis with focal hemophagocytosis. There was no evidence of metastatic carcinoma in the bone marrow aspirate and biopsy. Flow cytometry showed no immunophenotypic abnormalities. The patient fulfilled six out of eight diagnostic criteria for HLH: fever, splenomegaly, elevated ferritin level, pancytopenia, hypofibrinogenemia, and hemophagocytosis in the bone marrow (Figures 1, 2). 

After the diagnosis of HLH, the next step was to continue investigations to find out the underlying cause for HLH in this otherwise healthy patient. He underwent an extensive evaluation to look for secondary causes of HLH. Infectious workup including hepatitis panel, human immunodeficiency virus (HIV) antigen/antibody test, Lyme, babesia, anaplasmosis polymerase chain reaction (PCR), nasopharyngeal swab for influenza A and B, severe acute respiratory syndrome coronavirus 2 (SARS-CoV-2) nasopharyngeal swab, rapid PCR, IgG antibodies, gastrointestinal viral panel, leptospira antibody, West Nile virus IgM cytomegalovirus IgG/IgM returned negative. Autoimmune workup, including antinuclear antibody (ANA) titer, C3, C4 levels, was unremarkable. Flow cytometry of periphral blood was negative for monoclonal B-cell population or aberrant T-cell immunophenotype. Computed tomography (CT) of the abdomen pelvis revealed retroperitoneal lymphadenopathy. Unexpectedly, para-aortic lymph node biopsy revealed well-differentiated prostatic adenocarcinoma. After evaluation by a hematologist/oncologist, a rheumatologist, and an Infectious disease specialist, no other triggering factor for HLH was identified. Therefore, a diagnosis of HLH secondary to prostate adenocarcinoma was made. High dose steroids and etoposide were initiated for treatment of HLH. Soluble interleukin-2 receptor level (sCD25) was found to be elevated at 3.2 pg/mL (range <2.1 pg/mL), adding confirmation to the diagnosis of HLH. Unfortunately, despite high dose steroid treatment and chemotherapy with etoposide, his clinical status continued to deteriorate. The patient’s course was complicated by acute liver failure, disseminated intravascular coagulation, and upper gastrointestinal bleeding. He also developed tumor lysis syndrome 48 hours after initiating etoposide. His clinical course was rapid, and the patient died from multiorgan failure within two weeks from symptom onset.

An autopsy was performed to see if there was another undiagnosed concomitant pathology as the patient’s rapid clinical course was unusual and prostate cancer has only been reported three times in association with HLH in the literature. The autopsy report showed prostatic acinar adenocarcinoma, Gleason Score 4+5 = 9 (Grade Group 5) with seminal vesicle invasion and perineurial invasion. There were matted para-aortic lymph nodes/mass with metastatic prostate adenocarcinoma measuring 12.5 cm x 3.5 cm x 3.0 cm. There was a mediastinal lymph node with metastatic prostate adenocarcinoma as well. Bone marrow examination revealed many macrophages showing hemophagocytosis, which was consistent with the previous bone marrow biopsy report. Splenomegaly and hepatomegaly were noted with massive congestion and macrophage infiltration. No other pertinent pathology was identified on the detailed autopsy report, and it was concluded that the patient died from multiple organ failure caused by HLH secondary to prostatic adenocarcinoma.

## Discussion

HLH is characterized by prolonged fever, hepatosplenomegaly, cytopenia, hypertriglyceridemia, hyperferritinemia, and hemophagocytosis in bone marrow, liver, spleen, or lymph nodes [1]. The data regarding HLH in the setting of solid tumors are limited. A few solid tumors in the literature have been reported to be associated with HLH, which include breast cancer, thyroid carcinoma, and to our knowledge, this is the fourth report of secondary HLH in association with prostatic adenocarcinoma [4]. Declerck et al. described a patient with metastatic prostate cancer who developed a major hematoma secondary to pancytopenia caused by HLH while on chemotherapy. However, his HLH was successfully treated with vincristine and docetaxel [5]. Koizumi et al. described a case of HLH secondary to disseminated carcinomatosis in the setting of prostate cancer [6]. El Khoury et al. described a patient with metastatic prostate carcinoma who developed HLH in the setting of *Escherichia coli* sepsis. His clinical course improved with the treatment of sepsis with antibiotics [7].

The diagnosis of HLH needs thorough evaluation because the presentation is nonspecific. Due to the rarity of this disease, knowledge gaps have frequently resulted in misdiagnosis or delayed diagnosis. There have been reports where patients experienced prolonged hospitalization or clinical deterioration without a clear diagnosis before the possibility of HLH was raised [8,9].

HLH may simulate several conditions that cause fever, pancytopenia, liver function test abnormalities, or neurologic findings. The diagnosis is challenging because the presentation is nonspecific. Cytopenias, a very high ferritin level, and liver function abnormalities are especially helpful in distinguishing HLH from other conditions [10]. Both sepsis and HLH can have findings of disseminated intravascular coagulation and widespread inflammation with cytokine abnormalities. Sepsis is typically not characterized by ongoing lymphocyte activation. Extremely high ferritin, elevated lactate dehydrogenase level and negative blood cultures were highly suggestive of HLH rather than sepsis [11]. Macrophage activation syndrome was also considered a form of HLH associated with rheumatologic disease [12]. The patient was evaluated by the rheumatologist for evaluation of possible underlying rheumatological etiology. ANA, perinuclear antineutrophil cytoplasmic antibody (p-ANCA), cytoplasmic ANCA (c-ANCA), and anti-glomerular basement membrane (anti-GBM) antibody returned negative and thus rheumatological etiology was unlikely. TTP, hemolytic uremic syndrome (HUS), or drug-induced thrombotic microangiopathy (DITMA) can present with fever, anemia, neurologic findings, renal failure, and liver failure, but they generally do not have rising ferritin level and anemia is microangiopathic showing schistocytes on peripheral smear [13]. Primary liver disease and HLH can present with both hepatomegaly and elevated liver function tests. Both can cause coagulopathy with prolonged PT and activated partial thromboplastin time (aPTT), low fibrinogen, and elevated D-dimer, and can cause encephalopathy. Unlike liver disease, HLH is a multisystem disorder and typically has more extensive organ involvement, cytopenias, extremely high ferritin, and neurologic findings.

The diagnosis of HLH is based on fulfilling the published diagnostic criteria used in the HLH-2004 trial as below. In adults, heterozygosity of verified HLH-associated mutations or gene defects of other immune regulatory genes together with clinical findings associated with HLH or five of the following eight findings: fever ≥ 38.5°C; splenomegaly; peripheral blood cytopenia; hypertriglyceridemia (fasting triglycerides > 265 mg/dL); hypofibrinogenemia (fibrinogen < 150 mg/dL); hemophagocytosis in bone marrow, spleen, lymph node, or liver; low or absent natural killer cell activity; ferritin > 500 ng/mL; and elevated soluble CD25 (soluble IL-2 receptor alpha) are used to diagnose HLH [10]. Because of the high mortality of HLH in the absence of appropriate treatment, it is not always required to meet the criteria in order to initiate treatment. Specifically, treatment should not be delayed while awaiting the results of genetic or specialized immunologic testing.

In our patient, a diagnosis of HLH was made as he met six out of the eight criteria, after ruling out the differential diagnosis discussed above. The initial treatment is given based on the HLH-94 protocol in addition to the treatment of the causative disease. HLH-94-based therapy includes etoposide and dexamethasone given at tapering doses over eight weeks [14]. Therapy should not be delayed while awaiting specialized immunologic testing or genetic analysis. The prognosis of malignancy-associated HLH remains poor. A review of outcomes in adult HLH patients from three centers reported a 31% response rate with an overall survival of four months, but only three months in those with malignancy-associated HLH [15].

## Conclusions

HLH should be suspected in a patient with unexplained fever, hepatosplenomegaly, cytopenias, hepatitis, or central nervous system findings. The diagnosis is challenging as the patients may present with a clinical picture indistinguishable from sepsis or multiple organ dysfunction syndrome. A priority should be placed on rapid evaluation including a CBC with differential, coagulation studies, serum ferritin, liver function tests, triglycerides, blood cultures, and viral testing. The bone marrow should be examined for the cause of cytopenias, infectious organisms, hemophagocytosis, and macrophage infiltration. We would like to emphasize the importance of early diagnosis, initiation of a broad diagnostic workup, and the necessity of new therapeutic approaches to improve the prognosis of HLH in the future.
